# Obsessive–compulsive disorder post-COVID-19: a case presentation

**DOI:** 10.1186/s41983-021-00405-1

**Published:** 2021-10-30

**Authors:** Abdulmajeed A. Alkhamees

**Affiliations:** Department of Medicine, Unayzah College of Medicine and Medical Science, Qassim University, Al Qassim, Saudi Arabia

**Keywords:** COVID-19, Pandemic, OCD

## Abstract

**Background:**

Since the emergence of the COVID-19 pandemic and the significant changes that impacted because of it, people around the world have been left dealing with its consequences—fear of becoming ill and dying, fear of losing loved ones, uncertainty about the future, and imposed social isolation—several elements which could lead to psychological consequences. Moreover, as suggested by recent evidence, the virus acts as a factor in causing psychological symptoms, including depression, anxiety, fatigue, and post-traumatic stress disorder.

**Case presentation:**

Here, we report a case of a patient with new-onset OCD after his recovery from the coronavirus disease, which presented in the form of recurrent and persistent intrusive thoughts and doubts which responded to medication.

**Conclusions:**

This case shows the potential of COVID-19-associated inflammatory triggers to precipitate or induce obsessive–compulsive symptoms. Although this case cannot support causation, it does stress the bidirectional effects that physical and mental illness share.

## Background

Since the emergence of the COVID-19 pandemic and the significant changes that it caused, people around the world have been left dealing with its consequences—fear of becoming ill and dying, fear of losing loved ones, uncertainty about the future, and the imposed social isolation—several elements which could lead to psychological consequences. Moreover, recent evidence has suggested that the virus acts as a factor in causing psychological symptoms, including depression, anxiety, fatigue, and post-traumatic stress disorder [1].

Obsessive–compulsive disorder (OCD) has been of interest in the recent pandemic because it focuses on hand hygiene. It is characterized by the presence of obsessions and/or compulsions. Obsessions are recurring and constant thoughts, feelings, desires, or images that are perceived as distracting and unwelcome. Compulsions are repeated actions or behavioral activities that a person is forced to conduct in reaction to an urge or according to strict, self-imposed rules.

While the obsession and compulsion content differ between people, certain themes are common in OCD, including those of symmetry, forbidden or taboo thoughts, and harm. The prevalence of OCD is around 2% worldwide. Males and females are affected equally, with a bimodal age at onset (before the age of 20 in most cases and earlier in males); onset after age 50 is relatively rare and may be more likely to have an organic etiology. The exact cause of OCD is unknown, but mostly thought to be multifactorial; the interaction between genetic and environmental factors is suspected in the etiology.

A recent study investigated the prevalence of OCD during the COVID-19 pandemic in a Canadian Province [2] and concluded that the prevalence of OCD increased during the current COVID-19 pandemic at a rate significantly higher than the pre-pandemic rate. Moreover, during the surge of COVID-19 pandemic, an Egyptian study conducted on the general population and health care workers reported that incidence of anxiety and OCD were 29.5% and 28.2%, respectively [3]. Also a recent systematic review examining OCD during the current pandemic showed that obsessive–compulsive symptoms worsened during the early stages of the pandemic [4]. Additionally, there have been reports associating the incidence of OCD after infections [5], but no case was reported after the recent COVID-19 disease.

Here, we report a case of a patient with late-onset OCD after his recovery from the coronavirus disease

## Case

Mr. A is a 62-year-old male, married, retired, healthy, with no remarkable medical history. He was in his usual state of health until 2 months before his presentation in the clinic when he experienced an increase in body temperature with body ache, fatigue, and a dry cough. He went to his primary health care doctor, and a nose swab PCR test for COVID-19 virus was taken for him, which came back to be positive. He continued to manage his symptoms at home with only paracetamol and rest with no other medication, and all of his symptoms gradually subsided within 2 weeks. And on the third week, he repeated the nose swab PCR test, and the result came back negative. However, during the third and fourth week since the start of his symptoms, he started experiencing recurrent and persistent intrusive thoughts of doubts about his 20-year-old nephew being his own son. Even though he recognizes these thoughts as excessive and unreasonable, it makes him distressed, and in response to these thoughts, he would phone his nephew to ask about him indirectly. These thoughts were not going away and started to increase over time and to be followed by other recurrent and persistent thoughts about different things in his life, mainly on the theme of “doubts”, for example, going back repeatedly to check the car locks. In response, he started isolating himself, avoiding any family gatherings. His daily routine was disturbed. The patient denied having persistent and excessive worries about these thoughts; he denied having sudden periods of intense fear of dying or any other significant anxiety symptoms; he had no other significant mood symptoms nor apparent psychotic symptoms; the patient had no previous psychiatric history and no history of psychiatric illness in the family and the patient had no apparent cognitive impairment or changes before or after the infection. He presented to the clinic 2 months after the disappearance of his COVID-19 symptoms and the emergence of these new thoughts. A physical and neurological examination was performed with unremarkable findings. The patient had normal blood and chemistry results, including normal liver and renal function tests, but he never underwent a CT brain imaging. A formal diagnosis of obsessive–compulsive disorder with good insight was made.

He was started on escitalopram 10 mg daily and was referred for cognitive–behavioral psychotherapy and was given an appointment after 4 weeks.

In the fourth week, he self-reported that the recurrent and persistent intrusive thoughts were decreasing in intensity by approximately 60% on a scale from 0 to 100, but he still had them from time to time; the dose of escitalopram was increased to 20 mg, and he was given another appointment after 1 month at which he reported that most of these thoughts had disappeared and he started resuming his routine and daily living activity. Unfortunately, he dropped out of his psychotherapy sessions after the second visit, so he was advised to continue using the medication and was given a follow-up appointment.

## Discussion

This case shows the potential of COVID-19-associated inflammatory triggers to precipitate or induce obsessive–compulsive symptoms. The role of this virus in developing these symptoms needs to be understood. Several studies have suggested that OCD is associated with low-grade inflammation, especially autoantibodies directed against the basal ganglion [6]. In some cases of OCD, autoimmunity may be triggered by an infectious agent, which could be the case in our patient. Moreover, in a recent study that screened 402 adults surviving COVID-19 for psychiatric symptoms, 20% of patients self-reported obsessive–compulsive symptoms [7]. Similarly, a study in Egypt examined the long-term impact of COVID-19 infection on sleep and mental health reported the non-severe COVID-19 patients had the highest percentage in many psychiatric illnesses, including obsessive–compulsive and depression [8]. In our case, the patient responded to the lowest dose of escitalopram within 1 month, despite being offered psychotherapy, which he quit early in the session, as his symptoms started to fade.

The etiology of the psychiatric sequela of infection with coronavirus is likely to be from multiple factors and might include cerebrovascular disease, the immunological response, the direct effects of viral infection (including brain infection), medical interventions, social isolation, the psychological burden of being infected with a newly contagious virus, and concerns about spread and stigma.

Unfortunately, a limitation to this case report is that no scale was used to track the patient improvement and reduction in the severity of his symptoms, and we only relied mainly on clinical judgment, also imaging studies, and further workup was not done due to the patient’s refusal to go through additional workup, so it is hard to exclude the other organic factors in play in the development of these symptoms or to make an association between the COVID-19 infection and obsessive–compulsive disorder.

Nevertheless, it is worth noting that when the obsession is mentioned during the current pandemic, it is usually associated with contamination and its effect on worsening the already existing obsessive symptoms or making it difficult to comply with the whole precaution. Here, in our case, we shed light on another area that might be missed while evaluating patients after being infected with COVID-19.

## Recommendations

This case probably only shows the tip of the iceberg. Recent studies indicate an increase in the incidence of OCD symptoms in the general population tie, presumably related to coexistence with high-stress levels during the COVID-19 pandemic [9]. Thus, health workers should pay attention to early obsessive–compulsive symptoms and a quick approach in dealing with this with cognitive therapy or medication; although this case cannot support causation, it does stress the bidirectional effects that physical and mental illness share.

## Conclusions

We present a case of a 62-year-old male who presented with symptoms of a new-onset obsessive–compulsive disorder after having been diagnosed with COVID-19 infection.

Although this case cannot support causation, it does stress the bidirectional effects that physical and mental illness share.

## Data Availability

Data-sharing does not apply to this article as no new data were created or analyzed in this study.

## Abbreviations

COVID-19: Coronavirus disease 2019
OCD: Obsessive–compulsive disorder
